# Altered Actinobacteria and Firmicutes Phylum Associated Epitopes in Patients With Parkinson’s Disease

**DOI:** 10.3389/fimmu.2021.632482

**Published:** 2021-07-02

**Authors:** Zhuo Li, Gang Lu, Zhe Li, Bin Wu, Enli Luo, Xinmin Qiu, Jianwen Guo, Zhangyong Xia, Chunye Zheng, Qiaozhen Su, Yan Zeng, Wai Yee Chan, Xianwei Su, Qiaodi Cai, Yanjuan Xu, Yingjun Chen, Mingbang Wang, Wai Sang Poon, Xiaodong Luo

**Affiliations:** ^1^ Genetic Testing Lab, The Second Affiliated Hospital of Guangzhou University of Chinese Medicine, Guangzhou, China; ^2^ Division of Neurosurgery, Department of Surgery, Prince of Wales Hospital, The Chinese University of Hong Kong, Hong Kong, Hong Kong; ^3^ The Chinese University of Hong Kong-Shandong University (CUHK-SDU) Joint Laboratory on Reproductive Genetics, School of Biomedical Sciences, The Chinese University of Hong Kong, Hong Kong, Hong Kong; ^4^ Department of Neurology, The Second Affiliated Hospital of Guangzhou University of Chinese Medicine, Guangzhou, China; ^5^ Department of Neurology, Liaocheng People’s Hospital, Liaocheng, China; ^6^ Department of Neurology, Liaocheng Clinical School of Shandong First Medical University, Liaocheng, China; ^7^ The Second Clinical College of Guangzhou University of Chinese Medicine, Guangzhou, China; ^8^ Children’s Hospital of Fudan University, National Center for Children’s Health, Shanghai, China

**Keywords:** Parkinson’s disease, metagenome-wide association study, microbiota-associated epitopes, immunity, glutamate and propionate metabolism, Actinobacteria phylum, Firmicutes phylum

## Abstract

Recent evidence suggests that inflammation was participated in the pathogenesis of PD, thus, to understand the potential mechanism of gut microbiota in the pathogenesis of Parkinson’s disease (PD), we performed a metagenomic analysis of fecal samples from PD patient and controls. Using a two-stage metagenome-wide association strategy, fecal DNA samples from 69 PD patients and 244 controls in three groups (comprising 66 spouses, 97 age-matched, and 81 normal samples, respectively) were analyzed, and differences between candidate gut microbiota and microbiota-associated epitopes (MEs) were compared. In the study, 27 candidate bacterial biomarkers and twenty-eight candidate epitope peptides were significantly different between the PD patients and control groups. Further, enriched 4 and 13 MEs in PD were positively associated with abnormal inflammatory indicators [neutrophil percentage (NEUT.1), monocyte count/percentage (MONO/MONO.1), white blood cell count (WBC)] and five candidate bacterial biomarkers (c_Actinobacteria, f_Bifidobacteriaceae, *g_Bifidobacterium*, o_Bifidobacteriales, p_Actinobacteria) from Actinobacteria phylum, and they were also positively associated with histidine degradation and proline biosynthesis pathways, respectively. Additionally, enriched 2 MEs and 1 ME in PD were positively associated with above inflammatory indicators and two bacteria (f_Lactobacillaceae, *g_Lactobacillus*) from Firmicutes phylum, and they were also positively associated with pyruvate fermentation to propanoate I and negatively associated with isopropanol biosynthesis, respectively. Of these MEs, two MEs from GROEL2, RPSC were derived from *Mycobacterium tuberculosis*, triggered the T cell immune response, as previously reported. Additionally, other candidate epitope peptides derived from *Mycobacterium tuberculosis* and *Mycobacterium leprae* may also have potential immune effects in PD. In all, the altered MEs in PD may relate to abnormalities in immunity and glutamate and propionate metabolism, which furthers our understanding of the pathogenesis of PD.

## Introduction

Parkinson’s disease (PD) is one of the most common neurodegenerative disorders worldwide, for which there is currently no complete cure (1). Besides its motor and cognitive symptoms, many patients also have gastrointestinal (GI) dysfunction preceding other symptoms, such as constipation and an extended time interval between defecations (2). A study using transgenic mice carrying human A53T-α-synuclein (α-syn) found that while signs of GI dysfunction were observed in the mice at 3 months of age, motor abnormalities and α-syn pathology were only observed 6 months later (3).

The increasing research interest in the gut–brain axis has led to many in-depth studies of the pathology and gut microbiota in PD. Several studies have indicated an association between GI inflammation and the accumulation of α-syn in the enteric nervous system of PD (4). Transplantation of the gut microbiota from PD-affected patients enhances motor impairments and inflammation, with inflammation possibly posing as a risk factor for PD (5). Furthermore, a number of studies have indicated that bacterial infection is also associated with a higher risk of neurodegeneration. For instance, it has been shown that Bacillus Calmette–Guérin vaccine can induce neuroprotection in a mouse model of PD (6). In addition, pathogenic genes involved in PD, such as *PARK2*, *PINK1*, and *LRRK2*, are thought to regulate innate immunity and relate to susceptibility to bacterial infection in the gut. These results indicate that the effect of dysfunction of the gut microbiota on the immune system plays an important role in PD (7).

An increasing number of findings confirm that gut microbiota-associated epitopes (MEs) do affect the immune system, contributing to abnormal inflammatory reactions and the pathological status of neurological diseases; thus, their relationship with PD needs to be further studied. Additionally, gut microbiota may also affect the nervous system *via* the metabolites it releases. Certain metabolites such as glutamate can function as excitatory neurotransmitters and cause excitatory responses (8, 9). To further investigate the alteration of the gut microbiota and its potential effects on immunity and metabolism in PD, we employed a two-stage shotgun metagenomic approach and analyzed the data using the Immune Epitope Database (IEDB) (10), according to previously developed methods (11). Therefore, in the present study, we recruited 69 PD patients, 81 healthy controls (HC), 66 spouses of PD patients (SP), and 97 age-matched healthy controls (NG) from the public database, with the latter two groups included to minimize the effects of lifestyle/diet and age/gender, respectively. Based on the metagenome-wide association analysis, we identified the PD-associated gut microbiota and predicted MEs and pathways that may be responsible for effects on immune function and pathology in PD.

## Material and Methods

### Subjects

The protocols of this study were approved by the Research Ethics Committee at the Second Affiliated Hospital of Guangzhou University of Chinese Medicine. This study was registered in the China Clinical Trials Registry (ChiCTR) with registration number ChiCTR1800018493. Sixty-nine participants were enrolled from the Second Affiliated Hospital of Guangzhou University of Chinese Medicine from September 2018 to May 2019. Each participant was informed of the purpose of this study and signed a clearly stated consent form. All measurements were voluntary.

All patients (n = 69) were diagnosed with PD according to the diagnostic criteria proposed by the International Parkinson’s Disease and Movement Disorder Society in 2015 (12). Exclusion criteria for subjects with PD were as follows: (1) intake of probiotics or antibiotics within the last 3 months; (2) diagnosed with digestive system diseases such as inflammatory bowel disease, colitis, or colon cancer. Three control groups were used in this study. Firstly, the dataset for the HC group (n = 81) was included from another study (unpublished data). Secondly, the SP group (n = 66) was recruited as an internal control group to account for the influence of lifestyle and dietary factors on the gut microbiota. Thirdly, the dataset of an additional healthy control group, NG (n = 97), was included from a published database (13), and was used to account for the influence of age and gender. The same exclusion criteria were applied to the groups of healthy controls as were applied to the PD group.

### Clinical Data Collection

Clinical data were collected *via* face-to-face interviews. The weight and height of each participant were measured and their body mass index (BMI) was calculated. Questionnaires concerning non-motor symptoms, including the Non-Motor Symptoms Scale (NMSS), Hamilton Depression Scale (HAMD), Hamilton Anxiety Scale (HAMA), Montreal Cognitive Assessment (MoCA), Mini-Mental State Examination (MMSE) and MDS-Unified Parkinson’s Disease Rating Scale (MDS-UPDRS) were obtained from PD patients. In addition, 41 PD patients also received routine blood examination and assessment of the following inflammatory indicators: white blood cell count (WBC); neutrophil count/percentage (NEUT/NEUT.1); lymphocyte (LYM); monocyte count/percentage (MONO/MONO.1); eosinophilia (EOSIN); basophils (BASO); red blood cell count (RBC); hemoglobin (Hb); hematocrit (HCT); mean red blood cell volume (MCV); mean cell hemoglobin (MCH); mean cell hemoglobin concentration (MCHC); red blood cell distribution width (RDW); PLT; MPV; PCT; and platelet distribution width (PDW). All clinical data were listed in **Table 1**.

**Table 1:** Characteristics of the study subjects.

Characteristics	PD group (n = 69)	HC group (n = 81)	P value (PD, HC)	SP group (n = 66)	NG Group (n = 97)	P value (PD, NG)
Age (years)	58.1 (12.0)	36.3 (11.2)	<0.001	59.0 (12.1)	65.7(9.3)	0.608
Female (n, %)	28 (40.6)	14 (17.3)	<0.01	41 (62.1)	53 (54.6)	0.103
BMI (kg/m2)	23.1 (2.91)	23.3 (2.5)	0.919	23.2 (3.3)	–	–
H&Y stage	2.2 (0.55)	–	–	–	–	–
Age of onset (years)	58.8 (7.3)	–	–	–	–	–
Disease duration (years)	6.2 (4.5)	–	–	–	–	–
MDS_UPDRS	53.3 (27.0)	–	–	–	–	–
MDS_UPDRS_P1	8.0 (5.2)	–	–	–	–	–
MDS_UPDRS_P2	11.1 (7.2)	–	–	–	–	–
MDS_UPDRS_P3	29.0 (16.5)	–	–	–	–	–
MDS_UPDRS_P4	2.2 (3.2)	–	–	–	–	–
HAMA	10.1 (6.8)	–	–	–	–	–
HAMD	7.1 (5.2)	–	–	–	–	–
MoCA	23.7 (3.7)	–	–	–	–	–
NMSS	40.1 (26.2)	–	–	–	–	–
MMSE	27.7 (2.7)	–	–	–	–	–
WBC	6.3 (2.3)	–	–	–	–	–
NEUT.1	62.3 (12.6)	–	–	–	–	–
LYM	26.9 (10.6)	–	–	–	–	–
MONO.1	7.1 (2.7)	–	–	–	–	–
EOSIN	3.3 (5.3)	–	–	–	–	–
BASO	0.4 (0.3)	–	–	–	–	–
NEUT	4.1 (2.3)	–	–	–	–	–
LYM	1.6 (0.6)	–	–	–	–	–
MONO	0.4 (0.2)	–	–	–	–	–
EOSIN_COUNT	0.2 (0.4)	–	–	–	–	–
BASO	0.0 (0.0)	–	–	–	–	–
RBC	4.5 (0.5)	–	–	–	–	–
Hb	133.1 (11.2)	–	–	–	–	–
HCT	40.1 (3.2)	–	–	–	–	–
MCV	90.1(6.4)	–	–	–	–	–
MCH	29.9 (2.2)	–	–	–	–	–
MCHC	331.6 (8.8)	–	–	–	–	–
RDW	12.8 (0.7)	–	–	–	–	–
PLT	215.1 (56.6)	–	–	–	–	–
MPV	9.6 (1.2)	–	–	–	–	–
PCT	0.2 (0.0)	–	–	–	–	–
PDW	12.7 (2.6)	–	–	–	–	–

PD, Parkinson’s disease; HC, healthy control; SP, spouse of PD patients; NG, normal group; BMI, body mass index; H&Y stage, Hoehn and Yahr stage; MDS-UPDRS, MDS-Unified Parkinson’s Disease Rating Scale; MDS-UPDRS_PI, non-motor experiences of daily living; MDS-UPDRS_P2, motor experiences of daily living; MDS-UPDRS_P3, motor examination; MDS-UPDRS_P4, motor complications; HAMA, Hamilton Anxiety Scale; HAMD, Hamilton Depression Scale; MoCA, Montreal Cognitive Assessment; NMSS, Non-Motor Symptoms Scale; MMSE, Mini-Mental State Examination; WBC, white blood cell count; NEUT/NEUT.1, neutrophil count/percentage; LYM, lymphocyte; MONO/MONO.1, monocyte count/percentage; EOSIN, eosinophilia; BASO, Basophils; RBC, Red blood cell count; Hb, hemoglobin; HCT, Hematocrit; MCV, Mean red blood cell volume; MCH, mean cell hemoglobin; MCHC, mean cell hemoglobin concentration; RDW, Red blood cell distribution width; PLT, platelet count; MPV, mean platelet volume; PCT, plateletocrit; PDW, platelet distribution width. “-”; not available. Numbers are expressed as mean ± standard deviation. P < 0.05; significant differences; P > 0.05; no differences.

### Fecal Sample Collection, DNA Extraction, and Sequencing

Fecal DNA samples were typically collected at home using a QIAamp DNA Stool Mini Kit (Qiagen, Hilden, Germany) and transferred to our hospital within 48 h and stored at -20°C. A total of 1 μg DNA per sample was used as the input material for the DNA sample preparations. Sequencing libraries were generated using a NEBNext Ultra DNA Library Prep Kit for Illumina (NEB, E7645S) according to the manufacturer’s recommendations, and index codes were added to attribute sequences to each sample. DNA concentration was measured using a Qubit double stranded (ds) DNA Assay Kit (Life Technologies, CA, USA) in the Qubit 2.0 Fluorometer (Life Technologies, CA, USA). The DNA sample was fragmented by sonication to a size of 350 bp. Samples were sequenced using 150 bp paired-end reads on an Illumina HiSeq platform, on average, approximately 6 Gb read bases were generated per sample (Novogene Bioinformatics Technology, Beijing, China).

### Raw Sequencing Data Preprocessing

Data from the whole metagenome were trimmed and human reads were filtered using KneadData software (https://bitbucket.org/biobakery/kneaddata) with the default parameters. Trimmomatic (trimmomatic-0.36-3; with the parameters: “SLIDINGWINDOW: 4:20 MINLEN: 50”) (14) was used to remove low-quality reads and adaptor sequences, while bowtie2 (parameters: “–very-sensitive –dovetail”) (15) was used to perform an alignment to the hg37 genome and remove host reads. The corresponding data download path is “http://huttenhower.sph.harvard.edu/kneadData_databases/Homo_sapiens_hg37_and_human_contamination_Bowtie2_v0.1.tar.gz”. The remaining reads were aligned to the IEDB (10) to perform prediction and analysis of potential epitope peptides and T cell antigenic epitopes, after which the gut microbiota-associated epitope profile was generated (11). MetaPhIAn2 software (16) (using the default settings) was used to generate a metagenomic taxonomic profile for further analysis. Functional and pathway profiling was performed with HUMANn2 (17) using the UniRef90 database with default settings.

### Bioinformatic and Statistical Analysis

R software (version 3.1.0) was used for statistical analysis. The R function “adonis” (from the “vegan” package) was used to perform a permutation test between clinical parameters and the metagenomic taxonomic profile, while the “metaMDS” function was used to perform NMDS analysis. The inverse Simpson diversity index was measured using the “diversity” function (from the “vegan” package). Group differences in diversity and phylum-level profiles were analyzed using the Wilcoxon rank-sum test and the “boxplot” function was used to plot the results. Results with P < 0.05 were considered statistically significant. PD- or HC-enriched microbiota were identified from the taxonomic profile, functional profile, and gut microbiota-associated epitope profile, using the following methods: (1) the linear discriminant analysis (LDA) effect size (LEfSe) algorithm was used to identify taxonomic biomarkers (with LDA > 2.5); (2) The DESeq2 package was used to identify potential functional biomarkers (with P < 0.05, False Discovery Rate (FDR) < 0.05, and |deseq2.logfoldchange| > 0.58); (3) Epitope peptides are used to analyze the composition of gut microbiota at the functional level to predict the underlying immune function composition of gut microbiota, and the DESeq2 package was used to identify epitope biomarkers (with P < 0.05, FDR < 0.05, and |deseq2.logfoldchange| > 1). The Pearson correlation between clinical parameters and candidate biomarkers was calculated using the “cor.test()” function. Results with P < 0.05 and |correlation| > 0.2 were considered statistically significant. The same method was also used to analyze the correlation among different candidate biomarkers, and a heatmap was used to plot the results. Additionally, the “lm” function was used to calculate the regression between each clinical parameter and each candidate biomarker.

## Results

### Basic Characteristics of the PD and Control Groups

**Figure 1:**
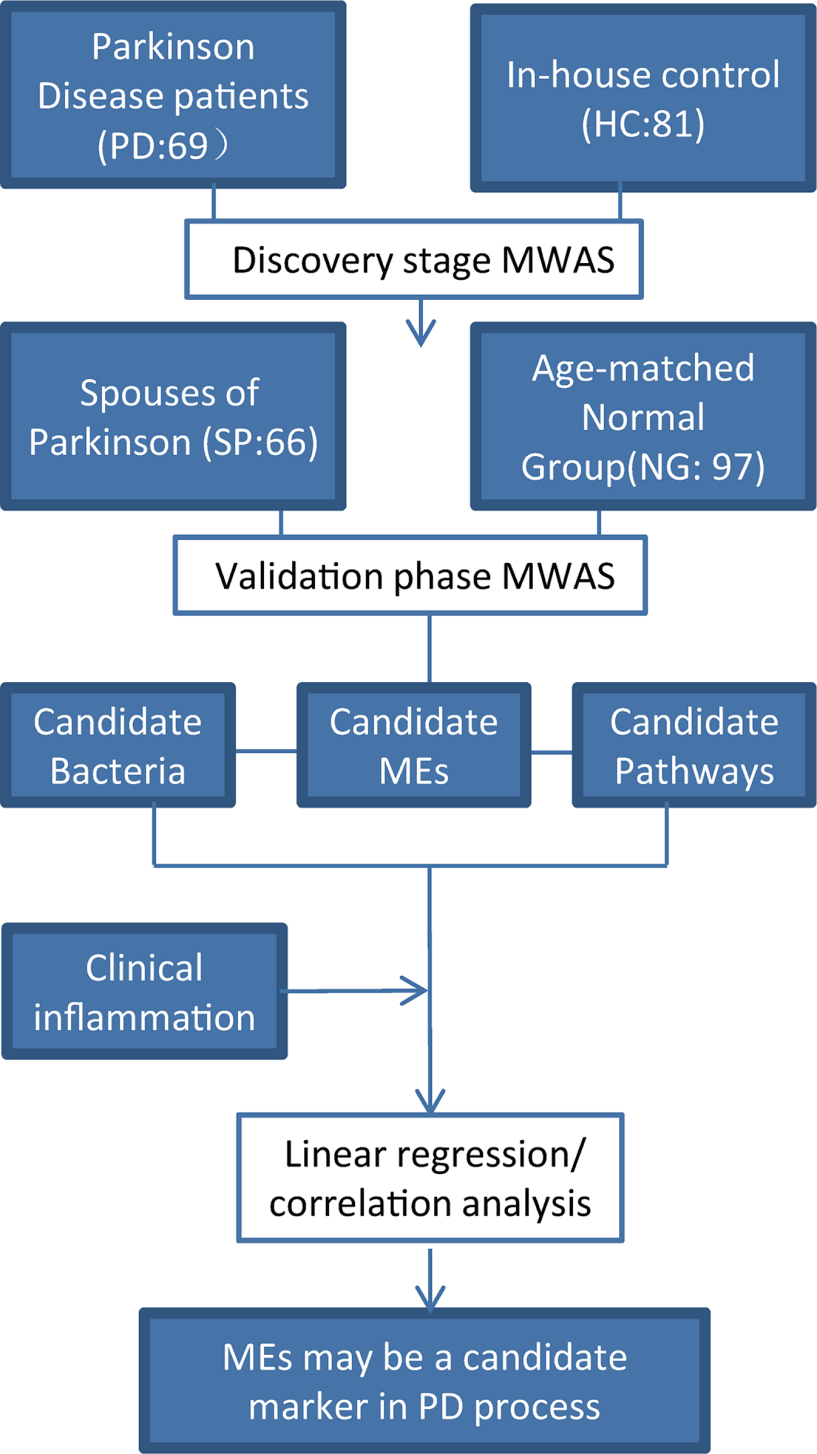
Study design. A two-stage (discovery and validate) MWAS analysis as used to identify potential bacterial biomarkers for PD. PD, Parkinson’s disease; SP, spouse of PD patients; NG, normal group; HC, healthy control. The SP group was used to account for the influence of lifestyle and dietary factors. The NG group was used to evaluate the effects of age and gender. Robust bacteria/epitope/pathway biomarkers were identified and their correlations with clinical parameters were evaluated.

A flow chart of the study design is shown (**Figure 1**). A total of 69 PD patients were recruited for the disease group, while 81 HC, 66 SP, and 97 NG subjects were recruited for the control groups. First, the PD group was compared with the HC group to screen for different gut microbiota between the two groups. Second, the SP group was used as the control group to eliminate the influence of environmental and dietary factors on gut microbiota. Finally, as significant differences in age and gender were detected between the PD and HC groups (P < 0.05), the NG group was included in this study to eliminate the influence of these two factors. The results showed that differences were not significant (P > 0.05) in the measured clinical indices in the PD and HC groups. The characteristics of the groups are summarized in **Table 1**.

### Metagenome-Wide Association Study and Identification of Potential Taxonomic Bacterial Biomarkers

**Figure 2:**
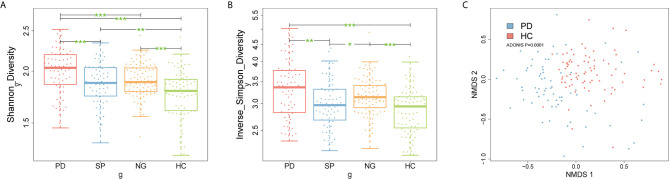
Microbial community differences between PD patients and control subjects. **(A, B)** Box plots describe differences in the microbiome diversity indices between PD and control groups according to the Shannon and inverse Simpson diversity indexes based on OTU levels. Each box plot represents the median, interquartile range, minimum, and maximum values. **(C)** Non-metric multidimensional scaling (NMDS) analysis of samples from PD group and HC group. Ordination based on Bray-Curtis dissimilarity calculated with genus-level data. Each dot represents one sample, the closer the dots are to one another, the more similar the microbiome compositions of these samples. *P < 0.05, **P < 0.01, ***P < 0.001 by the Wilcoxon rank-sum test. PD, Parkinson’s disease; SP, spouse of PD patients; NG, normal group; HC, healthy control; OTU, operational taxonomic unit.

To understand whether the composition of the gut microbiota differed between the PD and control groups, firstly, we compared the Shannon and inverse Simpson diversity indexes between groups. The results showed that gut microbiota diversity was significantly increased in patients with PD compared with that in the other control groups (**Figures 2A, B**, P < 0.05). Additionally, we further performed species-level non-metric multi-dimensional scaling (NMDS) analysis: the results showed that PD group was distinct from control samples (**Figure 2C**, **Supplementary Image 1**, P < 0.05). Meanwhile, there were 93 candidate bacterial biomarkers found in the metagenome-wide association study (MWAS) between the PD and HC groups. In contrast, for the SP and NG groups, we found that 66 candidate biomarkers were no longer significant, while the remaining 27 candidate biomarkers were considered to be important differential bacteria in PD. Among these, 12 robust bacterial biomarkers belonged to Actinobacteria phylum, 2 robust bacterial biomarkers belonged to Bacteroidetes phylum, 8 robust bacterial biomarkers belonged to Firmicutes phylum, and 5 robust bacterial biomarkers belonged to Proteobacteria phylum (**Supplementary Image 2**, P < 0.05). Furthermore, gender had a limited influence on these selected biomarkers, with no significant difference found between male and female samples (**Supplementary Image 3**, P > 0.05**)**. Taken together, our findings indicated that using multiple control groups can help to identify accurate PD-related biomarkers. A set of robust bacterial biomarkers were selected, which are listed in **Table S1** **(**FDR < 0.05**)**.

### Correlation Between Clinical Indicators in PD Patients and Candidate Bacterial Biomarkers

**Figure 3:**
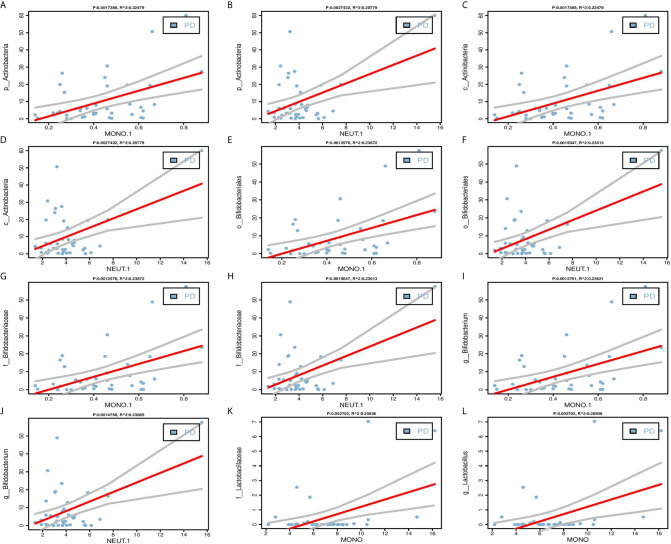
Linear regression between candidate biomarkers and inflammatory indicators. **(A–J)** Significant positive correlations between Actinobacteria phylum (p_Actinobacteria, c_Actinobacteria, o_Bifidobacteriales, f_Bifidobacteriaceae, *g_bifidobacterium*) and MONO.1, NEUT.1. **(K–L)** Significant positive correlations between Firmicutes phylum (f_Lactobacillaceae, *g_Lactobacillus*) and MONO. The Pearson correlation was calculated and tested using the “cor.test()” function. The linear regression analysis graph was plotted by the “lm” function.

One of the key functions of the gut microbiota is known to be its role in affecting the host’s immune system (18). Therefore, we performed linear regressions on PD patients data between each of the 27 selected bacterial biomarkers and inflammatory indicators. The results found that five candidate bacterial biomarkers (c_Actinobacteria, f_Bifidobacteriaceae, *g_Bifidobacterium*, o_Bifidobacteriales, p_Actinobacteria) belonged to Actinobacteria phylum have significant correlations with clinical indicators of inflammation including MONO.1 and NEUT.1, and two candidate bacterial biomarkers (f_Lactobacillaceae, *g_Lactobacillus*) belonged to Firmicutes phylum have significant correlations with MONO (**Figure 3**, **Table S2**, P < 0.05).

### Identification of MEs in the PD and Control Groups

**Figure 4:**
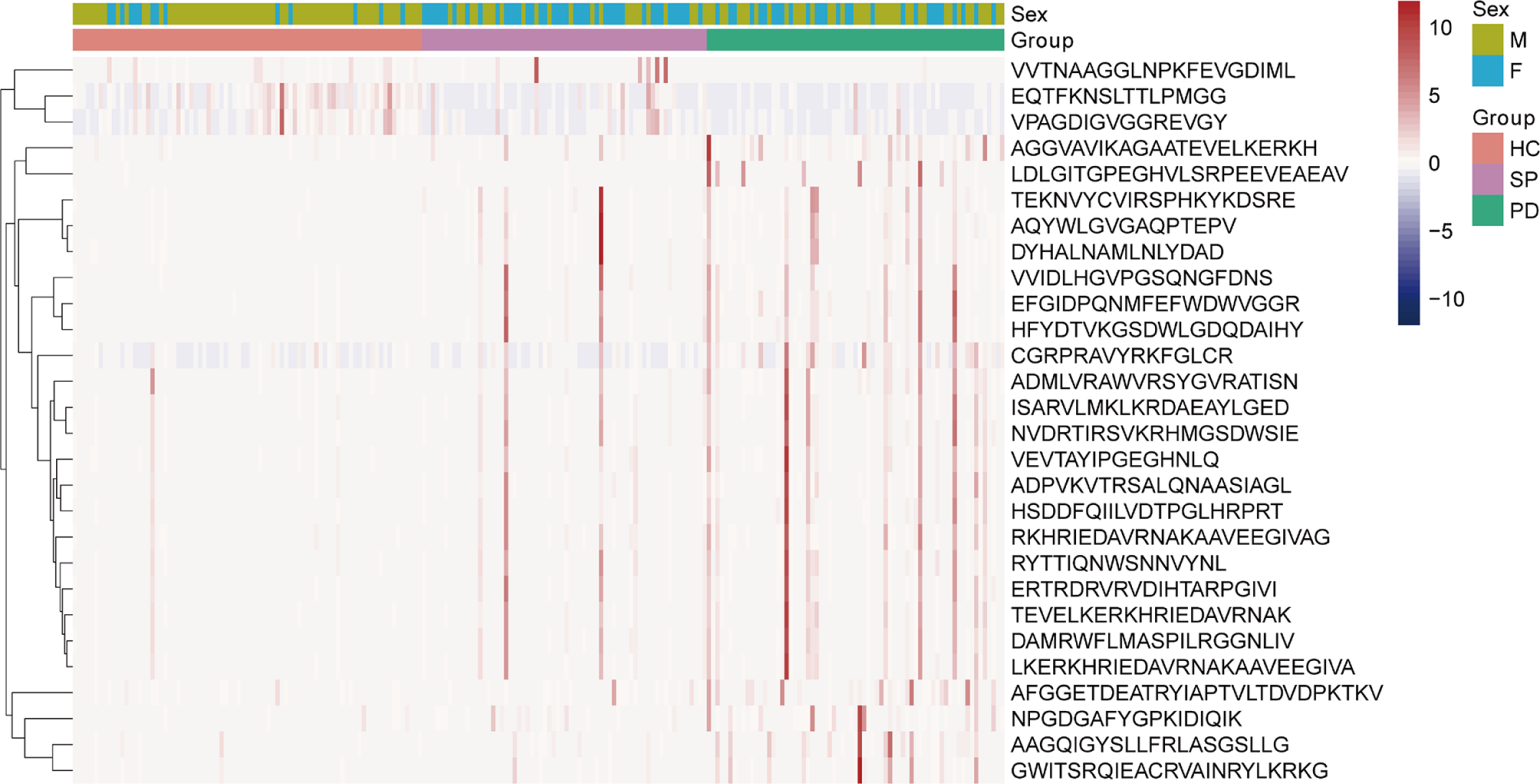
Analysis of gut microbiota-associated epitopes in PD patients and control subjects. Heatmap of significantly different epitope biomarkers in samples from PD group (Green), HC group (pink), SP group (purple). In the bar, pink and blue indicate high and low abundance, respectively.

To understand the possible immune functions of the gut microbiota, we predicted the composition of gut MEs in PD patients. A total of 28 significant epitope biomarker candidates were found, among which 25 were PD-enriched, while three were HC-enriched (**Figure 4**, FDR < 0.05). The PD-enriched MEs were very similar to peptides from proteins, for examples, one ME was derived from 50S ribosomal protein L16 (RPLP) from *Chlamydia trachomatis*; one ME from threonine–tRNA ligase 1, cytoplasmic (TARS1), derived from *Mus musculus* (mouse); one ME from cellulase domain-containing protein (PADG_07615), derived from *Paracoccidioides brasiliensis*; and one ME from 30S ribosomal protein S16 (RPSP), derived from *Mycolicibacterium smegmatis*. Furthermore, there were four MEs derived from *Homo sapiens* (human): one from glucose-6-phosphate isomerase (GPI); one from dihydropyrimidinase-related protein 2 (DPYSL2); one from succinate dehydrogenase [ubiquinone] flavoprotein subunit, mitochondrial (SDHA); and one from aldehyde dehydrogenase family 3 member A2 (ALDH3A2). In addition, there were five MEs derived from *Mycobacterium leprae:* three from 65-kD antigen (RML65); and two from chaperone protein DnaK (DNAK). There were 10 MEs derived from *Mycobacterium tuberculosis*, namely: one from 30S ribosomal protein S14 type Z (RPSZ); one from ribonucleoside-diphosphate reductase subunit alpha (NRDE); one from UPF0051 protein Rv1461 (RV1461); one from malate dehydrogenase (MDH); one from dTDP-glucose 4,6-dehydratase (RMLB); one from isoleucine–tRNA ligase (ILES); one from 30S ribosomal protein S3 (RPSC); one from 30S ribosomal protein S10 (RPSJ); one from 30S ribosomal protein S12 (RPSL); one from GTPase Era (ERA); and two from 60-kDa chaperonin 2 (GROEL2). Finally, the three HC-enriched MEs were also similar to peptides from proteins: one ME from purine nucleoside phosphorylase (PNP), derived from *Rattus norvegicus* (rat); and two MEs from glutamate dehydrogenase (GDH), derived from *Trypanosoma cruzi.* The distribution and origin of all epitope peptides are shown in **Table S3**.

### Gut MEs Correlate With Inflammatory Cytokines and Actinobacteria/Firmicutes Phylum Bacteria in PD Patients

**Figure 5:**
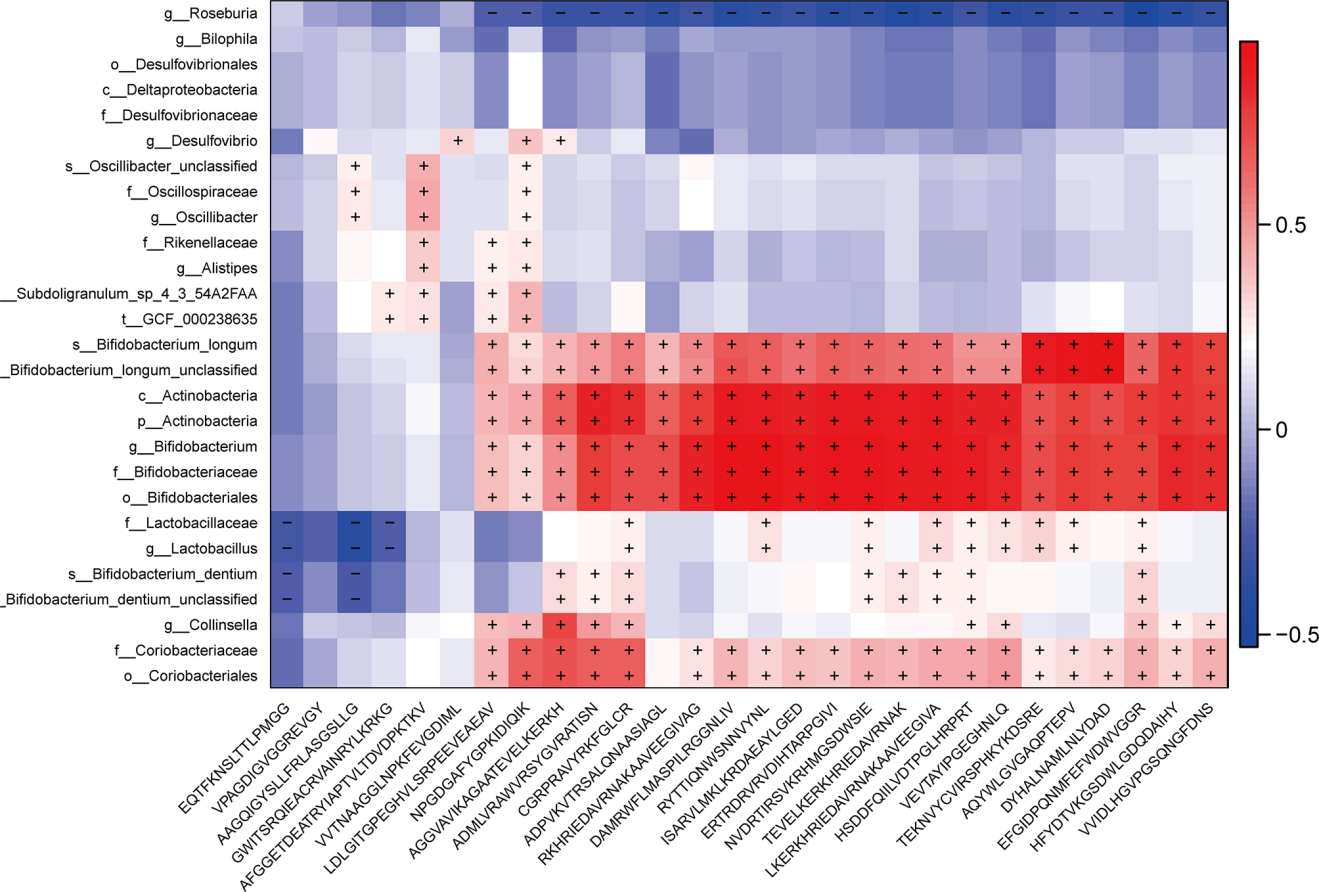
Correlations between 27 potential bacterial biomarkers and 28 selected epitope biomarkers. The Spearman correlation was calculated and tested using the “cor.test” function. The right panel shows the size of the correlation coefficient, with: red representing a positive correlation; blue representing a negative correlation; and “+” and “−” in each lattice representing significant positive and negative correlations (P < 0.05), respectively.

In order to investigate the relationship between differential MEs and gut microbiota composition or key clinical inflammatory indicators, we performed the linear regression between the 28 potential epitope biomarkers and inflammatory indicators and further evaluated the Spearman correlation between the selected epitope biomarkers and 27 bacterial biomarkers. The results showed that 16 potential epitopes enriched in PD were associated with MONO.1, NEUT.1 and WBC and also significantly positively correlated with above 5 Actinobacteria phylum candidate biomarkers (**Figure 5**, **Table S4**, P < 0.05**)**, and 6 potential epitopes enriched in PD were associated with MONO.1, NEUT.1 and also positively correlated with above two Firmicutes phylum candidate bacteria (**Figure 5**, **Table S5**, P < 0.05**).**

### Functional Pathways Correlate With Actinobacteria/Firmicutes Phylum Associated MEs

**Figure 6:**
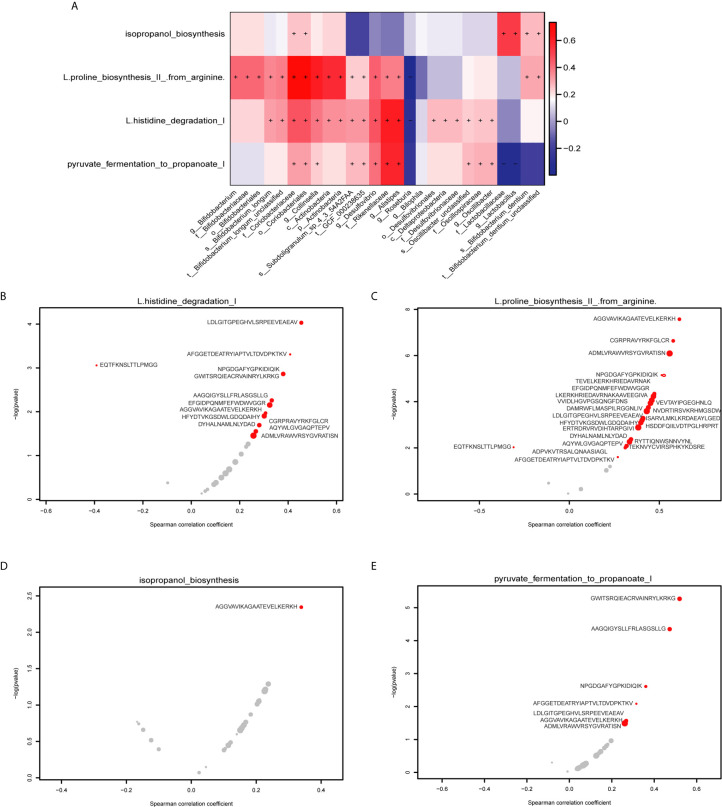
Correlations between gut MES and functional pathways. **(A)** The heatmap shows the correlations between 27 potential bacterial biomarkers and functional pathways. The right panel shows the size of the correlation coefficient, with: red representing a positive correlation; blue representing a negative correlation, “+” and “−” in each lattice representing significant positive and negative correlations, respectively (P < 0.05). **(B)** Correlations between the L-histidine degradation I pathway and 13 specific epitope biomarkers. **(C)** Correlations between the L-proline biosynthesis II (from arginine) pathway and 23 specific epitope biomarkers. **(D)** Correlations between the isopropanol biosynthesis pathway and 1 specific epitope biomarker. **(E)** Correlations between the pyruvate fermentation to propanoate I pathway and 7 specific epitope biomarkers. The horizontal axis represents the spearman correlation coefficient, the vertical axis represents -log(pvalue), the red dot represents the marker with significant correlation, and the gray represents the insignificant. The size of the dot represents the relative abundance of the marker.

We performed HUMAnN2 to identify significant functional pathways for gut microbiota in the PD group. The following four potential pathway biomarkers were found to be increased: L-histidine degradation I; pyruvate fermentation to propanoate I; isopropanol biosynthesis; and L-proline biosynthesis II (from arginine) (**Figure 6A**, P < 0.05). For five bacteria belonged to Actinobacteria phylum, the corresponding results showed that c_Actinobacteria, f_Bifidobacteriaceae, *g_Bifidobacterium*, o_Bifidobacteriales, and p_Actinobacteria was positively correlated with L-proline biosynthesis II (from arginine), and c_Actinobacteria, and p_Actinobacteria was also positively correlated with L-histidine degradation I. Additionally, for two bacteria belonged to Firmicutes phylum, f_Lactobacillaceae was positively correlated with pyruvate fermentation to propanoate I, and *g_Lactobacillus* was negatively correlated with isopropanol biosynthesis. Meanwhile, significant correlations between these four pathways and potential MEs were performed, and the results showed that 13 MEs, 23MEs, 1ME, and 7MEs were significantly correlated with the L-histidine degradation I, L-proline biosynthesis II (from arginine), isopropanol biosynthesis, and pyruvate fermentation to propanoate I, respectively (**Figures 6B–E**, **Table S6**, P < 0.05). Further, we found that 4/13 and 13/23 MEs were belonged to 16 MEs that correlated with the inflammation indicators and these five bacteria from Actinobacteria phylum, and 1/1 ME and 2/7 MEs were belonged to 6 MEs that correlated with the inflammation indicators and two bacteria from Firmicutes phylum (**Table 2**, P < 0.05).

**Table 2:** Actinobacteria/Firmicutes phylum and inflammatory markers associated epitopes in PD were significantly correlated with pathways enriched in PD.

PD Group	Pathways	MEs	Inflammatory biomarkers	Proteins	P value	Correlation	From
Actinobacteria phylum	L.histidine degradation I	LDLGITGPEGHVLSRPEEVEAEAV	MONO	DPYSL2	9.36E-05	0.452806	*Homo sapiens*
		CGRPRAVYRKFGLCR	NEUT.1, WBC	RPSZ	0.020310	0.278889	*Mycobacterium leprae*
		AGGVAVIKAGAATEVELKERKH	MONO	RML65	0.010781	0.305162	*Mycobacterium tuberculosis*
		ADMLVRAWVRSYGVRATISN	MONO.1, NEUT.1, WBC	RMLB	0.035289	0.253884	
	L.proline biosynthesis II from arginine	LDLGITGPEGHVLSRPEEVEAEAV	MONO	DPYSL2	0.00025	0.427394	*Homo sapiens*
		NVDRTIRSVKRHMGSDWSIE	NEUT.1, WBC	DNAK	0.000259	0.426357	*Mycobacterium leprae*
		AGGVAVIKAGAATEVELKERKH	MONO	RML65	2.69E-08	0.609702	*Mycobacterium tuberculosis*
		CGRPRAVYRKFGLCR	NEUT.1, WBC	RPSZ	2.33E-07	0.575328
		AGGVAVIKAGAATEVELKERKH	MONO	RML65	2.69E-08	0.609702
		CGRPRAVYRKFGLCR	NEUT.1, WBC	RPSZ	2.33E-07	0.575328
		ADMLVRAWVRSYGVRATISN	MONO.1, NEUT.1, WBC	RMLB	8.21E-07	0.553281
		TEVELKERKHRIEDAVRNAK	NEUT.1, WBC	RML65	4.60E-05	0.470052
		DAMRWFLMASPILRGGNLIV	NEUT.1, WBC	ILES	0.000266	0.425705
		HSDDFQIILVDTPGLHRPRT	NEUT.1	ERA	0.000819	0.393644
		ERTRDRVRVDIHTARPGIVI	NEUT.1, WBC	RPSC	0.001324	0.378935
		RYTTIQNWSNNVYNL	NEUT.1, WBC	RV1461	0.005517	0.330683
		ADPVKVTRSALQNAASIAGL	NEUT.1, WBC	GROEL2	0.009855	0.30871
Firmicutes phylum	Isopropanol biosynthesis	AGGVAVIKAGAATEVELKERKH	MONO	RML65	0.00453	0.337799	*Mycobacterium tuberculosis*
	Pyruvate fermentation to propanoate I	LDLGITGPEGHVLSRPEEVEAEAV	MONO	DPYSL2	4.50E-05	0.265658	*Homo sapiens*
		ADMLVRAWVRSYGVRATISN	MONO.1, NEUT.1, WBC	RMLB	5.42E-06	0.257598	*Mycobacterium tuberculosis*

## Discussion

In this study, we found 27 candidate bacterial biomarkers for PD, of which 26 were increased while only one (*Roseburia*) was decreased in the PD group (**Table S1**). Among these biomarkers, six have been reported in earlier studies. Five of these six previously reported bacterial biomarkers are consistent with our results, namely *Roseburia* (19, 20), Bifidobacteriaceae (19, 21), *Bifidobacterium* (19, 20), Desulfovibrionaceae (21), and Lactobacillaceae (20). The remaining one bacterial biomarker, *Lactobacillus*, was increased in the PD group in our results, which is consistent with results from some previous studies (20, 22), while not with those from two studies using Chinese patients (23). These contradictory findings might be attributable to regional and dietary differences in the PD patients and controls being studied. Our participants were mainly from South China, while the participants in the other two contradictory studies were from Northeast China and East China respectively (24). In addition to the biomarkers identified in the current study, a further 13 have been reported previously. However, these were not at the same taxonomic level as those described here, but were reported at either a higher or lower taxonomic level, including: *Alistipes* (25); Rikenellaceae (25); Deltaproteobacteria (21); Desulfovibrionales (21); *Bilophila* (21); *Desulfovibrio* (21); Actinobacteria (phylum and class) (19, 20); Bifidobacteriales (19, 20); *Bifidobacterium dentium* (19, 20); *Bifidobacterium longum* (19, 20); unclassified strain in *Bifidobacterium dentium* (19, 20); and unclassified strain in *Bifidobacterium longum* (19, 20). Nevertheless, we found that all 13 of these biomarkers were increased in the PD group, consistent with the results of earlier studies. The remaining eight biomarkers were identified for the first time in this study, comprising: Coriobacteriales; Coriobacteriaceae; *Collinsella*; Oscillospiraceae; *Oscillibacter*; unclassified species in *Oscillibacter*; *Subdoligranulum sp_4_3_54A2FAA*; and t-GCF_000238635. A previous report showed that *Collinsella* (related markers: Coriobacteriales and Coriobacteriaceae) was increased in other diseases, such as rheumatoid arthritis and type 2 diabetes (26), with a role for *Collinsella* in altering gut permeability and disease severity confirmed in experimental arthritis (27). *Oscillibacter* (related markers: Oscillospiraceae, *Oscillibacter*, and *unclassified species in Oscillibacter*) was reported to be related to diet-induced weight loss (28). *Subdoligranulum* (related markers: *Subdoligranulum sp_4_3_54A2FAA*, and t-GCF_000238635) was shown to be associated with constipation, a common symptom of PD. Taken together, these potential bacterial biomarkers may play important roles in PD, so the mechanisms through which these bacteria affect PD-related processes need to be further explored.

Intestinal dysfunction in PD may occur many years earlier than other symptoms, indicating that neurodegeneration may start in the gut and then begin later in the brain *via* the gut–brain axis. Gut microbiota can trigger the accumulation of α-synuclein and serve as a target for the diagnosis, treatment, and monitoring of disease progression in PD (29). Notably, a recent study showed that enteric inflammation plays an important role in PD pathogenesis (30). In the present study, we analyzed correlations between 27 gut microbiota biomarkers and clinical indicators in PD patients, and found a number of significant results. Interestingly, among the biomarkers from the four bacterial phyla, the highest abundance *Actinobacteria* phylum bacterial biomarkers and several biomarkers under this taxonomic level (c_Actinobacteria, f_Bifidobacteriaceae, *g_Bifidobacterium*, o_Bifidobacteriales) were positively correlated with inflammatory indicators, such as MONO, MONO.1, and NEUT, and f_Lactobacillaceae and *g_Lactobacillus* belonged to Firmicutes phylum were also positively correlated with MONO (**Table S2**, **Figure 3**). These results demonstrate that alterations to the gut microbiota and the inflammatory response play significant roles in the pathogenesis of PD. Previously, Wang et al. reported that the richness of MEs is associated with an elevated gut microbiota and gut Immunoglobulin A (IgA) level in patients with autism (11), which suggests that altered MEs may trigger abnormal immune function. In fact, more than 500000 MEs in the IEDB database are associated with adaptive immunity, which may cause a specific B-cell or T-cell response and participate in disease development. Therefore, in the present study, we identified that 25 MEs and three MEs were enriched in the PD and HC groups, respectively (**Table S3**, **Figure 4**). Of the PD-enriched MEs, 16 MEs were found related to inflammatory indicators and increased abundance of the 5 candidate biomarkers from Actinobacteria phylum, and 6 MEs were found related to inflammatory indicators and increased abundance of the 2 candidate biomarkers from Firmicutes phylum, suggesting that the gut microbiota may cause changes in the inflammatory response *via* alterations to MEs. This in turn may influence immune function and contribute to PD-related processes (**Tables S4** and **S5**).

Furthermore, to explore whether above 16 MEs that correlated with 5 bacteria from Actinobacteria phylum and inflammation markers were involved in PD pathogenesis mediated by the metabolic pathways, it was important to carry out a microbiome study at the functional level. The results showed that L-histidine degradation I and L-proline biosynthesis II from arginine enriched in the gut microbiota of the PD group were positively associated with 4 and 13 MEs, respectively, when comparing with the control group (**Table 2**). Histidine can be degraded to glutamate and thus cause changes in glutamate concentration in the cell. Additionally, glutamate and arginine are precursors to the formation of proline, so the metabolic pathway that promotes glutamate by arginine also further increases glutamate accumulation (31). In this study, these two enriched pathways may have indirectly caused an elevation in the concentration of glutamate and L-proline in the gut of PD patients, which is consistent with previous finding of PD disease (32). Glutamate functions as an excitatory neurotransmitter and can cause an excitatory response when it binds to its receptor, so the extracellular concentration of glutamate should remain low to maintain a healthy state (8). A higher extracellular concentration of glutamate can cause excitotoxicity, leading to malfunction of the neuron and cell death; this process has been associated with neurological and psychiatric diseases (8, 33). It is well known that inflammation can induce glutamate excitotoxicity (4). Interesting, among the MEs associated with these pathways, 11 of them are originated from *M. tuberculosis*, which has previously been associated with impaired innate defense mechanisms, chronic inflammation, and increased susceptibility to PD (34). In these MEs, two altered MEs have been found to be associated with immune function: ADPVKVTRSALQNAASIAGL from GROEL binds to human leukocyte antigen(HLA)-DR3 and restricts T cell responses (35); ERTRDRVRVDIHTARPGIVI from RPSC binds to major histocompatibility complex (MHC) class II, which is necessary for T cell recognition (36). Additionally, two of the significantly increased MEs are from *M. leprae*, an intracellular pathogen that commonly causes the chronic inflammatory disease leprosy. Recent research has found that *M. leprae* can invade nerve sheath cells, causing inflammatory reactions that lead to neuronal damage and neurodegeneration. The affinity of this pathogen for nerve cells increases the occurrence not only of leprosy, but potentially also of other neurological diseases such as PD, in which it may exert a crucial influence on PD-related pathogenesis (37). Moreover, the isopropanol biosynthesis pathway was significantly increased, while pyruvate fermentation to propanoate I pathway was significantly decreased in the gut microbiota of the PD group and both of them were positively associated with 1 ME and 2 MEs among the 6 MEs that correlated with two bacteria from Firmicutes and inflammation markers, respectively (**Table 2**). Isopropanol is an effective central nervous system inhibitor, which has a great influence on the brain (38). Propionic acid is one of the common short chain fatty acids, which can protect the integrity of intestinal mucosa (39). Thus, in the study, alteration of these two pathways may increase the isopropanol level and decrease the propionic acid production, which may cause damage to the nervous system and promote intestinal permeability leading to leaky intestines, respectively. A recent studies have shown that WBCs are key blood immune cells that release pro-inflammatory cytokines, and that neutrophils account for 50% to 70% of the total and are significantly associated with some clinical symptoms in PD (40). This is also similar to our studies, suggesting that peripheral blood inflammation plays a major role in the development of disease phenotypes. Thus, alteration of MEs may influence the immune responses in PD patients. However, although the three HC-enriched MEs were not correlated with the gut microbiota or with inflammation indicators, these peptides are also of interest: VVTNAAGGLNPKFEVGDIML from PNP binds to HLA-B7, while loss of PNP function can lead to severe T cell immunodeficiency or autoimmune disease (41). The other two peptides we found were from GDH, with the inhibition of GDH an important link between inflammation and excitotoxicity, which is also significantly associated with neurodegeneration in PD (42). Taken together, gut microbiota composition may produce epitopes, although correlations between some epitopes from these species and immunity has not been reported to date, the results of this study suggest that peptides from these proteins may play an important role in immunity in PD patients. This further indicates that the potentially dominant bacteria of the Actinobacteria phylum (c_Actinobacteria, f_Bifidobacteriaceae, *g_Bifidobacterium*, o_Bifidobacteriales, p_Actinobacteria) and Firmicutes phylum (f_Lactobacillaceae, *g_Lactobacillus*) may exert effects on immunity *via* these candidate MEs.

**Figure 7:**
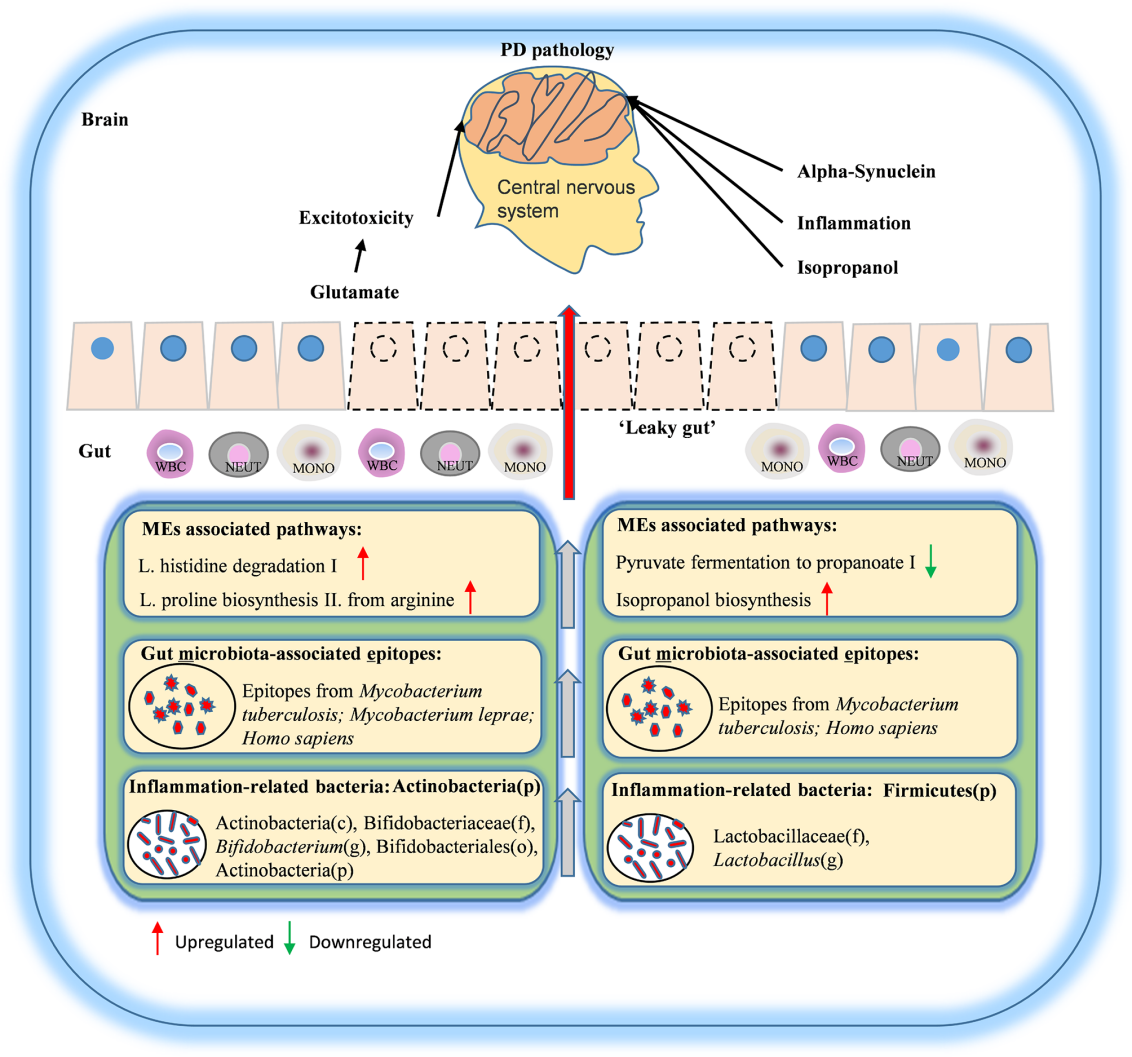
Schematic diagram of correlation of gut MEs with PD pathogenesis.

In all, in the present study, we analyzed a large number of samples from PD patients and healthy controls. We also recruited the SP and NG groups to minimize the influence of lifestyle factors, diet, gender, and age on the analysis of gut microbiota related to PD. Using various correlational and functional analyses, we found that altered MEs were significantly correlated with abnormal inflammation and biosynthesis of glutamate and propanoate. Thus, we proposed a model for gut MEs in PD pathogenesis according to these findings. We speculated that decreased abundance of propanoate may cause the increased intestinal permeability to deteriorate the normal gut permeability and generate “leaky gut”. Then inflammation and alpha-synuclein caused by the imbalance of the intestinal flora can affect the nerve function through the reverse transmission of the intestine-brain axis. In addition, glutamate may travel through the “leaky gut” and function as neurotransmitter to case excitotoxicity, and isopropanol may cause abnormal nervous system function (**Figure 7**). Taken together, these results suggested that altered intestinal MEs may aggravate the pathology of PD, and may be potential biomarkers of PD.

## Data Availability Statement

The datasets presented in this study can be found in online repositories. The names of the repository/repositories and accession number(s) can be found below: China National GeneBank [https://db.cngb.org/search/project/CNP0001697/].

## Ethics Statement

The studies involving human participants were reviewed and approved by the Ethics Committee of the Second Affiliated Hospital of Guangzhou University of Chinese Medicine. The patients/participants provided their written informed consent to participate in this study.

## Author Contributions

WP and XL conceived and designed the study. ZhuoL, GL, ZheL, and BW performed experiments and were major contributors in writing the manuscript, they were co-first authors. EL, XQ, JG, ZX, CZ, and XS made significant contributions to acquisition and analysis of data. QS, YZ, WC, XS, QC, YX, YC, and MW performed the statistical analysis. WP and XL revised the manuscript critically for important intellectual content. All authors contributed to the article and approved the submitted version.

## Funding

This work was supported by internal unrestricted research grant E473 to ZhuoL, Youth Program of National Natural Science Foundation of China (Grant No.: 82004459) and Natural Science Foundation of Guangdong Province, China (Grant No.: 2018A030310521).

## Conflict of Interest

The authors declare that the research was conducted in the absence of any commercial or financial relationships that could be construed as a potential conflict of interest.

## Supplementary Material

Supplementary Image 1 NMDS analysis of samples between PD group and SP group, PD group and NG group. Ordination based on Bray-Curtis dissimilarity calculated with genus-level data. Each dot represents one sample, the closer the dots are to one another, the more similar the microbiome compositions of these samples. PD, Parkinson’s disease; SP, spouse of PD patients; NG, normal group.

Supplementary Image 2 Potential bacterial biomarkers with altered abundance. Number of altered bacteria under different phyla after screening. 1_corrected, correction after using SP samples to eliminate environmental and dietary differences. 2_corrected, correction after using NG samples to eliminate age and gender differences.

Supplementary Image 3 Comparative analysis between potential bacterial biomarkers in males and females. No significant differences were found. SP_F, female in SP group; SP_M, male in SP group; HC_F, female in HC group; HC_M, male in HC group; NG_F, female in NG group; NG_M, male in NG group; PD_F, female in PD group; PD_M, male in PD group. *P < 0.05, **P < 0.01, ***P < 0.001 by the Wilcoxon rank-sum test.
