# Supplementary figures and images for: Altered Actinobacteria and Firmicutes Phylum Associated Epitopes in Patients With Parkinson’s Disease
Source: Front Immunol. 2021 Jul 2;12:632482. doi: 10.3389/fimmu.2021.632482 (PMC8284394; doi:10.3389/fimmu.2021.632482)

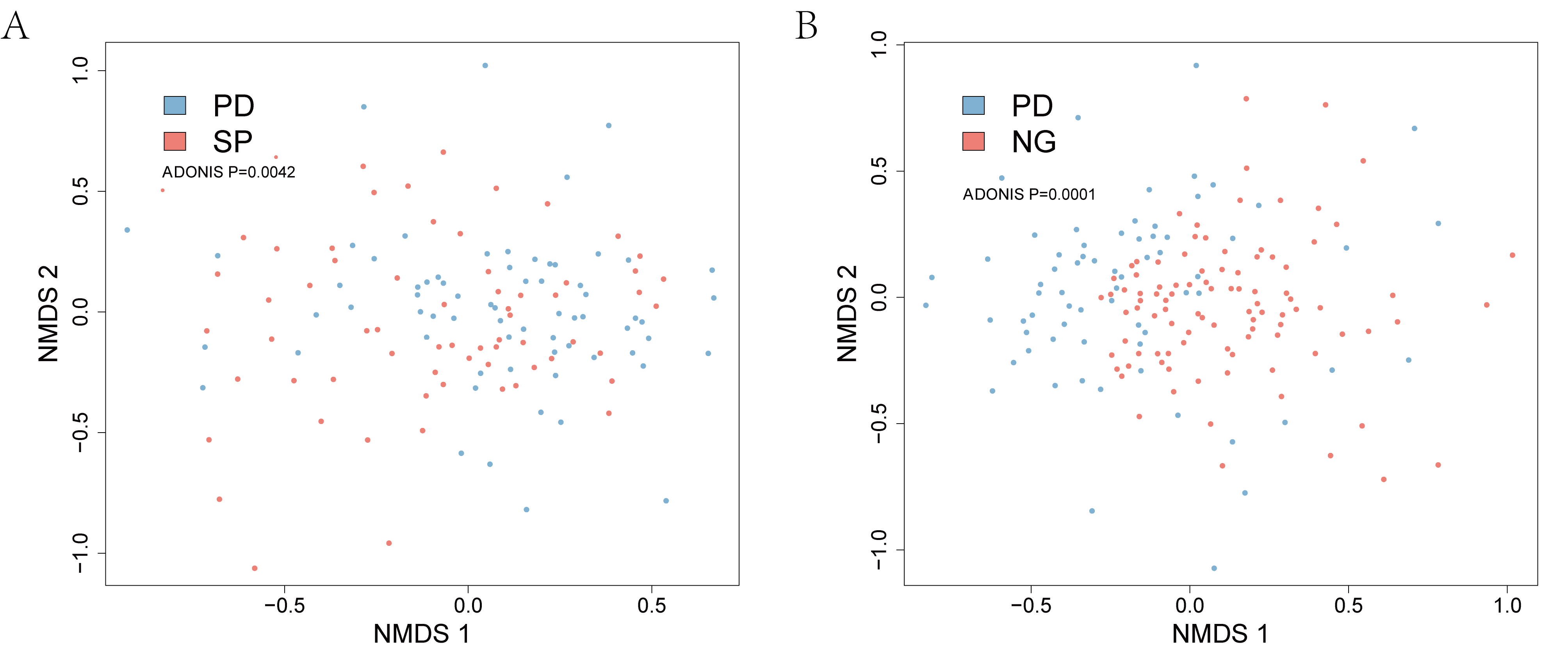

Supplement: Supplementary Image 1 — NMDS analysis of samples between PD group and SP group, PD group and NG group. Ordination based on Bray-Curtis dissimilarity calculated with genus-level data. Each dot represents one sample, the closer the dots are to one another, the more similar the microbiome compositions of these samples. PD, Parkinson’s disease; SP, spouse of PD patients; NG, normal group. [file Image_1.tif]

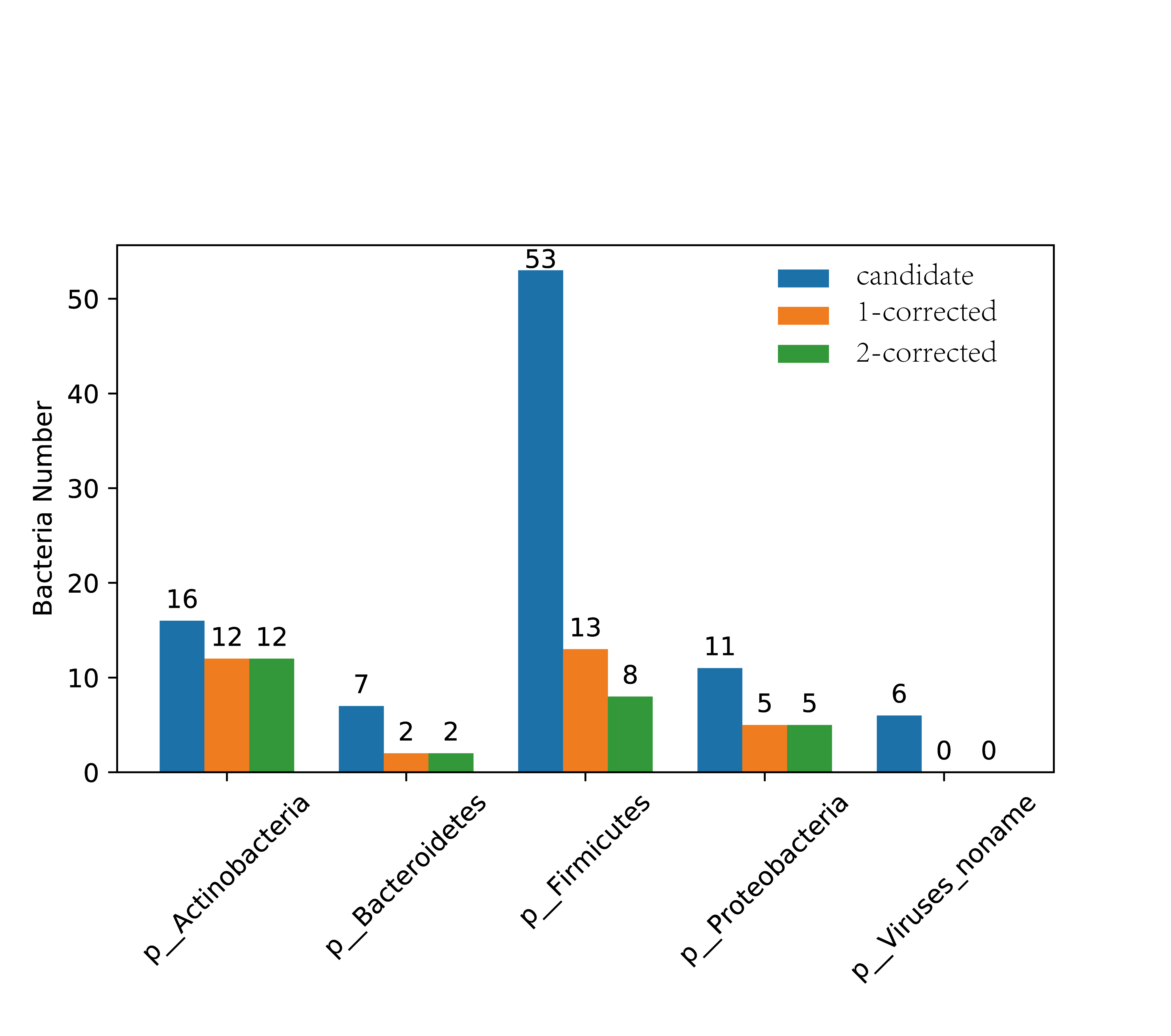

Supplement: Supplementary Image 2 — Potential bacterial biomarkers with altered abundance. Number of altered bacteria under different phyla after screening. 1_corrected, correction after using SP samples to eliminate environmental and dietary differences. 2_corrected, correction after using NG samples to eliminate age and gender differences. [file Image_2.tif]

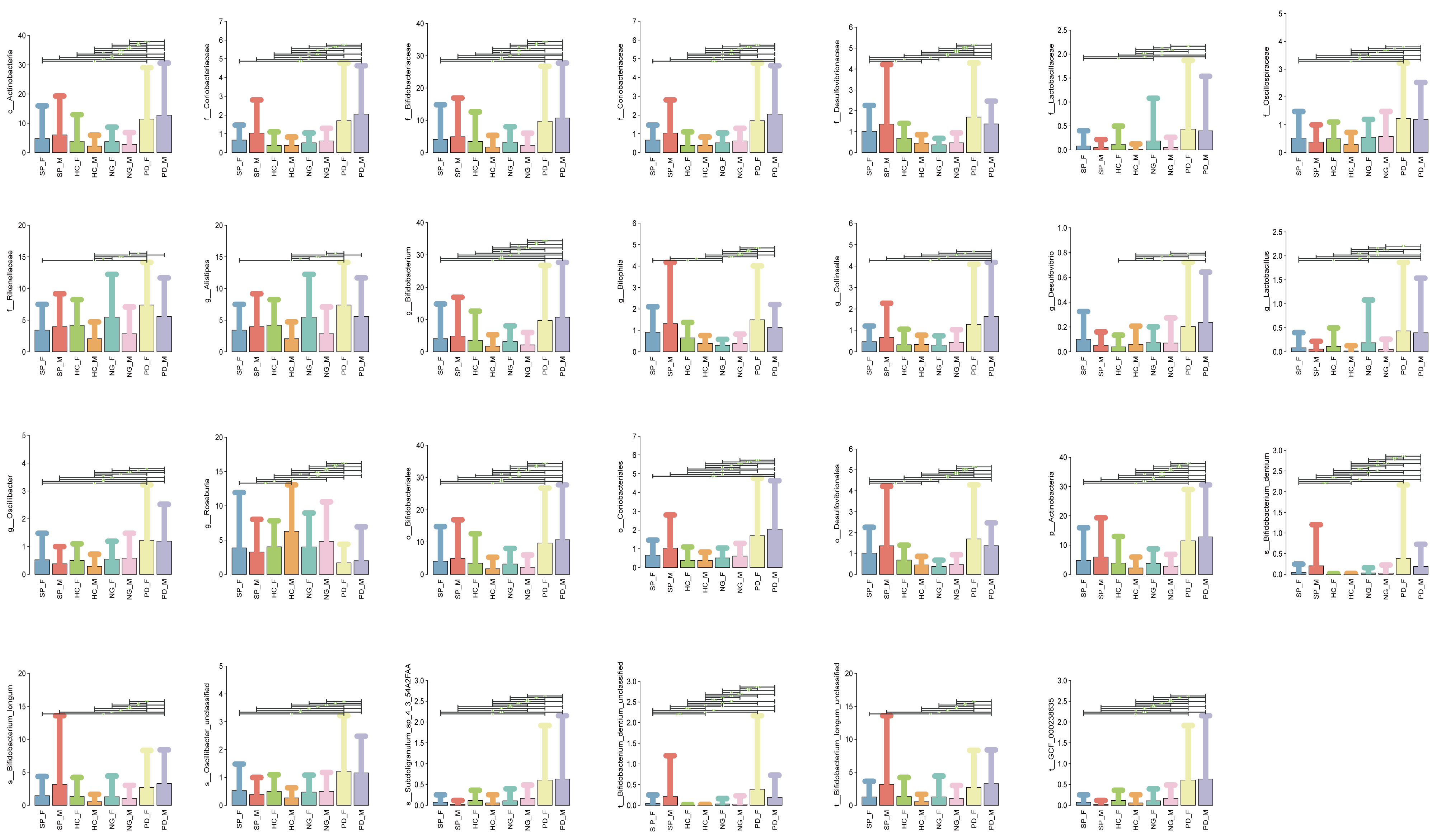

Supplement: Supplementary Image 3 — Comparative analysis between potential bacterial biomarkers in males and females. No significant differences were found. SP_F, female in SP group; SP_M, male in SP group; HC_F, female in HC group; HC_M, male in HC group; NG_F, female in NG group; NG_M, male in NG group; PD_F, female in PD group; PD_M, male in PD group. *P < 0.05, **P < 0.01, ***P < 0.001 by the Wilcoxon rank-sum test. [file Image_3.tif]
