# Supplementary material for: Altered Actinobacteria and Firmicutes Phylum Associated Epitopes in Patients With Parkinson’s Disease
Source: Front Immunol. 2021 Jul 2;12:632482. doi: 10.3389/fimmu.2021.632482 (PMC8284394; doi:10.3389/fimmu.2021.632482)
Supplement: Supplementary file 4 [file Table_1.doc]

**Table S1** Potential bacterial biomarkers with altered abundance

| **Group** | **Phylum** | **Taxonomy** | **Log Mean** | **Lda Score** | **P value** | **Other Studies** |
| --- | --- | --- | --- | --- | --- | --- |
| PD | Actinobacteria | Actinobacteria (class)^1^ | 5.09 | 4.58 | 1.92E-05 |  |
|  |  | Bifidobacteriaceae (family)^2^ | 5.01 | 4.51 | 4.06E-05 | [^1-3^](#_ENREF_1) |
|  |  | Coriobacteriaceae (family)^3^ | 4.28 | 3.74 | 0.001224 |  |
|  |  | *Bifidobacterium* (genus)^2^ | 5.01 | 4.51 | 4.38E-05 |  |
|  |  | *Collinsella* (genus)^3^ | 4.17 | 3.68 | 0.004358 |  |
|  |  | Bifidobacteriales (order)^1^ | 5.01 | 4.51 | 4.06E-05 |  |
|  |  | Coriobacteriales (order)^3^ | 4.28 | 3.74 | 0.001224 |  |
|  |  | Actinobacteria (phylum)^1^ | 5.09 | 4.58 | 1.92E-05 |  |
|  |  | *Bifidobacterium dentium* (species)^1^ | 3.43 | 2.95 | 0.001464 |  |
|  |  | *Bifidobacterium longum* (species)^1^ | 4.48 | 3.80 | 0.005533 |  |
|  |  | unclassified strain in *Bifidobacterium dentium* (strain)^1^ | 3.43 | 2.94 | 0.001464 |  |
|  |  | unclassified strain in *Bifidobacterium longum* (strain)^1^ | 4.48 | 3.83 | 0.006248 |  |
|  | Bacteroidetes | Rikenellaceae (family)^1^ | 4.80 | 4.09 | 0.018228 | [^4^](#_ENREF_4) |
|  |  | *Alistipes* (genus)^1^ | 4.80 | 4.09 | 0.018228 | [^4^](#_ENREF_4) |
|  | Firmicutes | Lactobacillaceae (family)^2^ | 3.62 | 3.26 | 0.003470 |  |
|  |  | Oscillospiraceae (family)^3^ | 4.08 | 3.57 | 9.51E-05 |  |
|  |  | *Lactobacillus* (genus)^4^ | 3.62 | 3.26 | 0.002336 |  |
|  |  | *Oscillibacter* (genus)^3^ | 4.08 | 3.57 | 9.51E-05 |  |
|  |  | *Roseburia* (genus)^2^ | 4.56 | 3.96 | 0.017760 |  |
|  |  | unclassified species in *Oscillibacter* (species)^3^ | 4.07 | 3.56 | 0.000118 |  |
|  |  | *Subdoligranulum sp_4_3_54A2FAA* (species)^3^ | 3.79 | 3.49 | 1.17E-05 |  |
|  |  | GCF 000238635 (strain)^3^ | 3.79 | 3.48 | 1.17E-05 |  |
|  | Proteobacteria | Deltaproteobacteria (class)^1^ | 4.18 | 3.33 | 0.014556 | [^3^](#_ENREF_3) |
|  |  | Desulfovibrionaceae (family)^2^ | 4.18 | 3.33 | 0.014735 | [^3^](#_ENREF_3) |
|  |  | *Bilophila* (genus)^1^ | 4.11 | 3.24 | 0.047091 | [^3^](#_ENREF_3) |
|  |  | *Desulfovibrio* (genus)^1^ | 3.35 | 2.80 | 0.049590 | [^3^](#_ENREF_3) |
|  |  | Desulfovibrionales (order)^1^ | 4.17 | 3.33 | 0.0147355 | [^3^](#_ENREF_3) |

Note: 1, Reported previously at the different taxonomic level, 2, Reported previously at the same taxonomic level. 3, Novel findings. 4, Inconsistent findings.
