# Supplementary material for: Altered Actinobacteria and Firmicutes Phylum Associated Epitopes in Patients With Parkinson’s Disease
Source: Front Immunol. 2021 Jul 2;12:632482. doi: 10.3389/fimmu.2021.632482 (PMC8284394; doi:10.3389/fimmu.2021.632482)
Supplement: Supplementary file 5 [file Table_2.docx]

**Table S2** Significant associations between seven candidate biomarkers and clinical indicators of inflammation.

| **Phylum** | **Taxonomy** | **Significant correlation** | **P value** | **Correlation** |
| --- | --- | --- | --- | --- |
| Actinobacteria | c_Actinobacteria | MONO.1 | 0.001739 | 0.224793 |
|  |  | NEUT.1 | 0.002743 | 0.207792 |
|  | f_Bifidobacteriaceae | MONO.1 | 0.001258 | 0.236718 |
|  |  | NEUT.1 | 0.001505 | 0.230131 |
|  | *g_Bifidobacterium* | MONO.1 | 0.001275 | 0.236211 |
|  |  | NEUT.1 | 0.001476 | 0.230847 |
|  | o_Bifidobacteriales | MONO.1 | 0.001258 | 0.236718 |
|  |  | NEUT.1 | 0.001505 | 0.230131 |
|  | p_Actinobacteria | MONO.1 | 0.001739 | 0.224793 |
|  |  | NEUT.1 | 0.002743 | 0.207792 |
| Firmicutes | f_Lactobacillaceae | MONO | 0.002702 | 0.208360 |
|  | *g_Lactobacillus* | MONO | 0.002702 | 0.208360 |
