# Supplementary material for: Altered Actinobacteria and Firmicutes Phylum Associated Epitopes in Patients With Parkinson’s Disease
Source: Front Immunol. 2021 Jul 2;12:632482. doi: 10.3389/fimmu.2021.632482 (PMC8284394; doi:10.3389/fimmu.2021.632482)
Supplement: Supplementary file 6 [file Table_3.docx]

**Table S3** Gut microbiota-associated epitopes enriched in the PD and HC groups

| **Group** | **Epitopes** | **PD.mean** | **PD.sd** | **HC.mean** | **HC.sd** | **P value** | **Protein** | **From** |
| --- | --- | --- | --- | --- | --- | --- | --- | --- |
| **PD** | AAGQIGYSLLFRLASGSLLG | 23.5942 | 51.48738 | 2.135802 | 7.729413 | 6.86E-08 | Malate dehydrogenase (MDH) | *Mycobacterium tuberculosis* |
|  | ADMLVRAWVRSYGVRATISN | 145.7101 | 270.1606 | 35.64198 | 107.2158 | 2.05E-06 | dTDP-glucose 4,6-dehydratase (RMLB) |  |
|  | ADPVKVTRSALQNAASIAGL | 9.42029 | 21.25573 | 1.308642 | 3.246698 | 1.27E-05 | 60 kDa chaperonin 2 (GROEL2) |  |
|  | CGRPRAVYRKFGLCR | 11.98551 | 16.70549 | 3.049383 | 5.007248 | 2.70E-06 | 30S ribosomal protein S14 type Z (RPSZ) |  |
|  | DAMRWFLMASPILRGGNLIV | 104.8261 | 225.077 | 16.07407 | 38.73234 | 1.85E-07 | Isoleucine--tRNA ligase (ILES) |  |
|  | DYHALNAMLNLYDAD | 26.76812 | 52.45142 | 4.185185 | 8.151857 | 5.29E-05 | Ribonucleoside-diphosphate reductase subunit alpha (NRDE) |  |
|  | ERTRDRVRVDIHTARPGIVI | 148.3478 | 316.0184 | 19.62963 | 47.66063 | 2.72E-08 | 30S ribosomal protein S3(RPSC) |  |
|  | HSDDFQIILVDTPGLHRPRT | 62.55072 | 141.2996 | 8.037037 | 23.6524 | 5.80E-07 | GTPase Era (ERA) |  |
|  | RYTTIQNWSNNVYNL | 76.18841 | 158.4522 | 12.48148 | 28.68585 | 6.92E-07 | UPF0051 protein Rv1461 (RV1461) |  |
|  | TEKNVYCVIRSPHKYKDSRE | 22.94203 | 46.49538 | 3.345679 | 5.484434 | 5.55E-06 | 60 kDa chaperonin 2 (GROEL2) |  |
|  | RKHRIEDAVRNAKAAVEEGIVAG | 10.72464 | 22.20088 | 1.82716 | 3.846395 | 4.64E-06 | 30S ribosomal protein S12 (RPSL) |  |
|  | VEVTAYIPGEGHNLQ | 84.50725 | 172.6454 | 22.50617 | 29.77882 | 0.00219 | 30S ribosomal protein S10 (RPSJ) |  |
|  | AGGVAVIKAGAATEVELKERKH | 11.56522 | 21.20313 | 2.740741 | 3.181107 | 0.000156 | 65 kd antigen (RML65) | *Mycobacterium leprae* |
|  | TEVELKERKHRIEDAVRNAK | 18.36232 | 41.11783 | 2.530864 | 6.734401 | 1.01E-07 | 65 kd antigen (RML65) |  |
|  | ISARVLMKLKRDAEAYLGED | 42.62319 | 99.46387 | 5.91358 | 16.91759 | 1.13E-08 | Chaperone protein DnaK (DNAK) |  |
|  | LKERKHRIEDAVRNAKAAVEEGIVA | 93.42029 | 195.6686 | 14.09877 | 26.69298 | 2.86E-09 | 65 kd antigen (RML65) |  |
|  | NVDRTIRSVKRHMGSDWSIE | 148.9275 | 315.0459 | 20.7284 | 54.31114 | 7.43E-08 | Chaperone protein DnaK (DNAK) |  |
|  | LDLGITGPEGHVLSRPEEVEAEAV | 18.26087 | 50.8613 | 0.296296 | 1.155903 | 5.66E-05 | Dihydropyrimidinase-related protein 2(DPYSL2) | *Homo sapiens* |
|  | EFGIDPQNMFEFWDWVGGR | 76.71014 | 162.2651 | 10.38272 | 16.24159 | 2.84E-06 | Glucose-6-phosphate isomerase (GPI) |  |
|  | HFYDTVKGSDWLGDQDAIHY | 55.84058 | 127.7765 | 5.91358 | 10.1897 | 5.65E-06 | Succinate dehydrogenase [ubiquinone] flavoprotein subunit, mitochondrial (SDHA) |  |
|  | AFGGETDEATRYIAPTVLTDVDPKTKV | 1.942029 | 3.522549 | 0.37037 | 0.872417 | 0.000611 | Aldehyde dehydrogenase family 3 member A2(ALDH3A2) |  |
|  | GWITSRQIEACRVAINRYLKRKG | 28.27536 | 77.60437 | 2.716049 | 6.492755 | 0.00333 | 50S ribosomal protein L16 (RPLP) | *Chlamydia trachomatis* |
|  | VVIDLHGVPGSQNGFDNS | 44.89855 | 91.99459 | 5.62963 | 10.93097 | 8.20E-06 | Cellulase domain-containing protein (PADG_07615) | *Paracoccidioides brasiliensis* |
|  | AQYWLGVGAQPTEPV | 28.4058 | 54.66161 | 3.037037 | 6.311585 | 3.30E-07 | 30S ribosomal protein S16 (RPSP) | *Mycolicibacterium smegmatis* |
|  | NPGDGAFYGPKIDIQIK | 6.289855 | 13.19436 | 0.765432 | 1.762609 | 2.02E-10 | Threonine--tRNA ligase 1, cytoplasmic (TARS1) | *Mus musculus* |
| **HC** | VPAGDIGVGGREVGY | 5.434783 | 11.2857 | 17.85185 | 24.87173 | 5.28E-07 | glutamate dehydrogenase (GDH) | *Trypanosoma cruzi* |
|  | EQTFKNSLTTLPMGG | 1.347826 | 2.299783 | 5.580247 | 5.071647 | 2.14E-12 | glutamate dehydrogenase (GDH) |  |
|  | VVTNAAGGLNPKFEVGDIML | 2.246377 | 5.389002 | 13.28395 | 22.59272 | 1.79E-07 | Purine nucleoside phosphorylase (PNP) | *Rattus norvegicus* |
