# Supplementary material for: Altered Actinobacteria and Firmicutes Phylum Associated Epitopes in Patients With Parkinson’s Disease
Source: Front Immunol. 2021 Jul 2;12:632482. doi: 10.3389/fimmu.2021.632482 (PMC8284394; doi:10.3389/fimmu.2021.632482)
Supplement: Supplementary file 7 [file Table_4.docx]

**Table S4** The enrichment of 16 selected epitopes in PD were significantly correlated with inflammatory markers and five candidate bacteria from Actinobacteria phylum

| **Group** | **MEs** | **Proteins** | **Inflammatory biomarkers** | **P value** | **Correlation** | **From** |
| --- | --- | --- | --- | --- | --- | --- |
| PD | ADMLVRAWVRSYGVRATISN | RMLB | MONO.1, | 0.0022 | 0.250474 | *Mycobacterium tuberculosis* |
|  |  |  | NEUT.1 | 2.88E-06 | 0.489789 |  |
|  |  |  | WBC | 8.11E-06 | 0.457811 |  |
|  | ADPVKVTRSALQNAASIAGL | GROEL2 | NEUT.1 | 6.72E-08 | 0.591643 |  |
|  |  |  | WBC | 2.97E-07 | 0.553987 |  |
|  | CGRPRAVYRKFGLCR | RPSZ | NEUT.1 | 3.65E-06 | 0.482674 |  |
|  |  |  | WBC | 3.84E-05 | 0.406115 |  |
|  | DAMRWFLMASPILRGGNLIV | ILES | NEUT.1 | 4.79E-08 | 0.599758 |  |
|  |  |  | WBC | 5.62E-07 | 0.536771 |  |
|  | ERTRDRVRVDIHTARPGIVI | RPSC | NEUT.1 | 2.78E-08 | 0.612531 |  |
|  |  |  | WBC | 2.16E-07 | 0.56229 |  |
|  | HSDDFQIILVDTPGLHRPRT | ERA | NEUT.1 | 1.05E-08 | 0.634343 |  |
|  |  |  | WBC | 6.30E-08 | 0.59321 |  |
|  | RYTTIQNWSNNVYNL | RV1461 | NEUT.1 | 1.19E-07 | 0.577465 |  |
|  |  |  | WBC | 1.56E-06 | 0.507977 |  |
|  | VEVTAYIPGEGHNLQ | RPSJ | NEUT.1 | 8.44E-07 | 0.525506 |  |
|  |  |  | WBC | 4.10E-06 | 0.479113 |  |
|  | RKHRIEDAVRNAKAAVEEGIVAG | **RPSL** | NEUT.1 | 4.33E-07 | 0.543872 |  |
|  |  |  | WBC | 4.40E-06 | 0.476967 |  |
|  | TEVELKERKHRIEDAVRNAK | RML65 | NEUT.1 | 5.57E-08 | 0.596166 |  |
|  |  |  | WBC | 8.05E-07 | 0.526847 |  |
|  | AGGVAVIKAGAATEVELKERKH | RML65 | MONO | 0.001277 | 0.273177 |  |
|  | ISARVLMKLKRDAEAYLGED | DNAK | NEUT.1 | 8.44E-07 | 0.525506 | *Mycobacterium leprae* |
|  |  |  | WBC | 4.10E-06 | 0.479113 |  |
|  |  |  | MONO.1 | 0.005001 | 0.2152 |  |
|  | LKERKHRIEDAVRNAKAAVEEGIVA | RML65 | NEUT.1 | 3.51E-08 | 0.607096 |  |
|  |  |  | WBC | 4.16E-07 | 0.544979 |  |
|  | NVDRTIRSVKRHMGSDWSIE | DNAK | NEUT.1 | 3.51E-07 | 0.549544 |  |
|  |  |  | WBC | 1.04E-06 | 0.519638 |  |
|  | LDLGITGPEGHVLSRPEEVEAEAV | **DPYSL2** | MONO | 0.003097 | 0.235926 | *Homo sapiens* |
|  | VVIDLHGVPGSQNGFDNS | PADG_07615 | NEUT.1 | 0.004331 | 0.221459 | *Paracoccidioides brasiliensis* |
