# Supplementary material for: Altered Actinobacteria and Firmicutes Phylum Associated Epitopes in Patients With Parkinson’s Disease
Source: Front Immunol. 2021 Jul 2;12:632482. doi: 10.3389/fimmu.2021.632482 (PMC8284394; doi:10.3389/fimmu.2021.632482)
Supplement: Supplementary file 8 [file Table_5.docx]

**Table S5** The enrichment of 6 selected epitopes in PD were significantly correlated with inflammatory markers and two candidate bacteria from Firmicutes phylum

| **Group** | **MEs** | **Proteins** | **Inflammatory biomarkers** | **P value** | **Correlation** | **From** |
| --- | --- | --- | --- | --- | --- | --- |
| PD | CGRPRAVYRKFGLCR | RPSZ | NEUT.1 | 3.65E-06 | 0.482674 | *Mycobacterium tuberculosis* |
|  |  |  | WBC | 3.84E-05 | 0.406115 |  |
|  | HSDDFQIILVDTPGLHRPRT | ERA | NEUT.1 | 1.05E-08 | 0.634343 |  |
|  |  |  | WBC | 6.30E-08 | 0.59321 |  |
|  | RYTTIQNWSNNVYNL | RV1461 | NEUT.1 | 1.19E-07 | 0.577465 |  |
|  |  |  | WBC | 1.56E-06 | 0.507977 |  |
|  | VEVTAYIPGEGHNLQ | RPSJ | NEUT.1 | 8.44E-07 | 0.525506 |  |
|  |  |  | WBC | 4.10E-06 | 0.479113 |  |
|  | NVDRTIRSVKRHMGSDWSIE | DNAK | NEUT.1 | 3.51E-07 | 0.549544 | *Mycobacterium leprae* |
|  |  |  | WBC | 1.04E-06 | 0.519638 |  |
|  | LKERKHRIEDAVRNAKAAVEEGIVA | RML65 | NEUT.1 | 3.51E-08 | 0.607096 |  |
|  |  |  | WBC | 4.16E-07 | 0.544979 |  |
