# Supplementary material for: Altered Actinobacteria and Firmicutes Phylum Associated Epitopes in Patients With Parkinson’s Disease
Source: Front Immunol. 2021 Jul 2;12:632482. doi: 10.3389/fimmu.2021.632482 (PMC8284394; doi:10.3389/fimmu.2021.632482)
Supplement: Supplementary file 9 [file Table_6.docx]

**Table S6** Significant correlation between gut MEs and enrichment pathways in PD

| **MEs** | **P value** | **Correlation** | **pathways** |
| --- | --- | --- | --- |
| LDLGITGPEGHVLSRPEEVEAEAV | 9.36E-05 | 0.452806 | L.histidine degradation I |
| AFGGETDEATRYIAPTVLTDVDPKTKV | 0.000495 | 0.408356 |  |
| EQTFKNSLTTLPMGG | 0.000877 | -0.391581 |  |
| NPGDGAFYGPKIDIQIK | 0.001323 | 0.378943 |  |
| GWITSRQIEACRVAINRYLKRKG | 0.001381 | 0.377604 |  |
| AAGQIGYSLLFRLASGSLLG | 0.005537 | 0.330551 |  |
| EFGIDPQNMFEFWDWVGGR | 0.007070 | 0.321482 |  |
| AGGVAVIKAGAATEVELKERKH | 0.010782 | 0.305163 |  |
| HFYDTVKGSDWLGDQDAIHY | 0.012576 | 0.298986 |  |
| CGRPRAVYRKFGLCR | 0.020311 | 0.278889 |  |
| DYHALNAMLNLYDAD | 0.020313 | 0.278884 |  |
| AQYWLGVGAQPTEPV | 0.028117 | 0.264431 |  |
| ADMLVRAWVRSYGVRATISN | 0.035289 | 0.253885 |  |
| AGGVAVIKAGAATEVELKERKH | 2.69E-08 | 0.609702 | L.proline biosynthesis II. From arginine |
| CGRPRAVYRKFGLCR | 2.33E-07 | 0.575328 |  |
| ADMLVRAWVRSYGVRATISN | 8.21E-07 | 0.553281 |  |
| NPGDGAFYGPKIDIQIK | 7.14E-06 | 0.511302 |  |
| TEVELKERKHRIEDAVRNAK | 4.60E-05 | 0.470052 |  |
| EFGIDPQNMFEFWDWVGGR | 5.91E-05 | 0.464081 |  |
| LKERKHRIEDAVRNAKAAVEEGIVA | 8.76E-05 | 0.454443 |  |
| VEVTAYIPGEGHNLQ | 0.000117 | 0.447137 |  |
| VVIDLHGVPGSQNGFDNS | 0.00018 | 0.436065 |  |
| LDLGITGPEGHVLSRPEEVEAEAV | 0.00025 | 0.427394 |  |
| NVDRTIRSVKRHMGSDWSIE | 0.000259 | 0.426357 |  |
| DAMRWFLMASPILRGGNLIV | 0.000266 | 0.425705 |  |
| ISARVLMKLKRDAEAYLGED | 0.000532 | 0.406302 |  |
| HFYDTVKGSDWLGDQDAIHY | 0.000641 | 0.400881 |  |
| HSDDFQIILVDTPGLHRPRT | 0.000819 | 0.393644 |  |
| ERTRDRVRVDIHTARPGIVI | 0.001324 | 0.378935 |  |
| DYHALNAMLNLYDAD | 0.004301 | 0.339645 |  |
| AQYWLGVGAQPTEPV | 0.005136 | 0.333286 |  |
| RYTTIQNWSNNVYNL | 0.005517 | 0.330683 |  |
| TEKNVYCVIRSPHKYKDSRE | 0.008334 | 0.315224 |  |
| EQTFKNSLTTLPMGG | 0.009469 | -0.31027 |  |
| ADPVKVTRSALQNAASIAGL | 0.009855 | 0.30871 |  |
| AFGGETDEATRYIAPTVLTDVDPKTKV | 0.025491 | 0.268867 |  |
| AGGVAVIKAGAATEVELKERKH | 0.00453 | 0.337799 | Isopropanol biosynthesis |
| ADMLVRAWVRSYGVRATISN | 5.42E-06 | 0.257598 | Pyruvate fermentation to propanoate l |
| LDLGITGPEGHVLSRPEEVEAEAV | 4.50E-05 | 0.265658 |  |
| NPGDGAFYGPKIDIQIK | 0.008303 | 0.358778 |  |
| GWITSRQIEACRVAINRYLKRKG | 0.026878 | 0.516968 |  |
| AFGGETDEATRYIAPTVLTDVDPKTKV | 0.032608 | 0.315368 |  |
